# Blurred magnitude homology of functional connectome for ASD diagnosis

**DOI:** 10.3389/fpsyt.2025.1677282

**Published:** 2026-01-08

**Authors:** Alexander Kachura, Vsevolod Chernyshev, Oleg Kachan, Egor Levchenko

**Affiliations:** 1 Faculty of Computer Science, HSE University (National Research University Higher School of Economics), Moscow, Russia; 2 Ulm University, Ulm, Germany; 3 Institute for Cognitive Neuroscience, HSE University (National Research University Higher School of Economics), Moscow, Russia

**Keywords:** blurred magnitude homology, persistent homology, functional connectivity, autism spectrum disorder, fMRI

## Abstract

Autism spectrum disorder (ASD) is one of the most common neurodevelopmental disorders. Existing studies show that adults with ASD may experience accelerated or altered neurocognitive aging. Consequently, cognitive decline in people with ASD can be delayed if timely measures are taken to treat this disorder. This study focuses on the development of a new algorithm for the early prediction of ASD from fMRI images. Autism spectrum disorder alters functional connectivity between brain regions. Therefore, it is important to develop methods for diagnosing this condition based on the analysis of a brain network. Functional brain networks are usually studied using undirected correlations, while functional connections in the brain are inherently directed. Blurred magnitude homology is an algebro-topological tool that enables the analysis of directed graphs, including directed functional connectomes. The method proposed in this work is based on applying a fully connected neural network to blurred magnitude homology-based features of a directed functional connectivity network. Experiments on empirically derived connectomes from fMRI images show that blurred magnitude homology is a useful invariant for distinguishing directed brain networks of individuals with ASD and typically developing individuals.

## 1 Introduction

Autism spectrum disorder (ASD) is one of the most prevalent neurodevelopmental disorders. The primary symptoms are difficulties in communication, repetitive behavior, and restricted interests. There is also evidence that adults with ASD may experience changes in cognitive functions related to aging that occur earlier than usual or follow a different trajectory than typically expected (see, for example, Dickinson et al. (1)Sigar et al. (2)). Therefore, early detection of this disorder is crucial, as it allows for timely intervention that not only significantly improves developmental outcomes, including communication, social skills, and overall adaptive behavior but may also potentially postpone cognitive decline.

Currently, ASD is diagnosed using the Diagnostic and Statistical Manual of Mental Disorders or analysis of scans obtained with neuroimaging technologies. Magnetic resonance imaging (MRI) is one of the most prevalent neuroimaging techniques. It helps observe structural brain changes that lead to the disorder. Functional MRI (fMRI) can provide information about the functional organization of brain networks and their association with cognitive functions (3). An fMRI scan is easy to collect during an MRI session. It defines biomarkers based on the brain’s functional organization that can be useful for the classification of ASD and may offer insights into the nature of the disease.

Existing approaches to fMRI-based ASD detection take as input the voxel or region of interest (ROI) time series directly or consider the model of the brain functional or effective connectome in the form of a connectivity matrix. The rationale for using brain networks to diagnose ASD is supported by evidence of altered connectivity in this condition as reported in many studies (4, 5). Given such a matrix, one can use it as an input directly by utilizing the off-diagonal or upper diagonal elements for the undirected or directed connectivity, respectively, or consider it as a graph, usually after a thresholding procedure.

Among the group of methods that work with fMRI data directly, Ahammed et al. considered all 3D images within a 4D fMRI sequence independently and applied a convolutional neural network (CNN) to them (6). Jiang et al. suggested aggregating the embeddings of separate 3D images from a 4D fMRI sequence obtained with a combination of a 3D-CNN and gated recurrent unit (GRU) (7). More recently, ASD diagnosis models have incorporated temporal-frequency analysis techniques to better capture dynamic brain activity patterns. Ke et al. proposed Fourier attention (8), and Wang et al. proposed wavelet attention models (9, 10), which exploit signal frequency dynamics in fMRI data to enhance diagnostic accuracy. Additionally, Wang et al. introduced a multimodal fusion approach combining generative adversarial networks and non-negative tensor decomposition to integrate multiple neuroimaging data sources (11).

For ASD diagnosis from connectomes, both methods that apply classical machine learning models to manually constructed features and deep learning approaches that work with connectomes directly exist. Popular graph features employed to solve the problem under consideration include node degree, node centrality measures, characteristic path length, clustering coefficient, efficiency, within-module degree z-score, participation coefficient, small-world propensity, and transitivity among others (see, for example, Chaitra et al. (12)Kazeminejad and Sotero (13)). The analysis of directed connectivity is performed less frequently. However, there are some studies in which authors used graph measures to study directed brain networks of patients suffering from neurodegenerative diseases—for example, to analyze connectivity patterns in Parkinson’s disease, Mijalkov et al. exploited features such as in-degree and out-degree, efficiency, diameter, transitivity, and clustering coefficient (14). There is also a study by Khazaee et al. on Alzheimer’s disease classification using node in-degree and out-degree, betweenness and pagerank centrality, flow coefficient, local and global efficiency, clustering coefficient, characteristic path length, transitivity, and assortativity (15). Deep learning approaches for ASD diagnosis are usually applied to undirected connectomes as well—for example, Eslami et al. proposed training an autoencoder on a vectorized adjacency matrix and an MLP (multi-layer perceptron) classifier obtaining the output of the encoder part of the autoencoder as an input simultaneously (16). There are also several graph neural networks that have been proposed to solve the problem of automatic detection of ASD—for example, Liu et al. suggested using a graph attention network with rows of a connectome adjacency matrix in the role of node features (17). Li et al. developed an ROI community assignment-based convolution layer and a new pooling layer selecting important nodes for a graph convolutional network (18). Experiments with transformer-based neural architectures were also conducted—for example, Kan et al. suggested using a multi-head self-attention module to construct embeddings of brain network nodes and transform node-level embeddings to graph-level ones via soft clustering through orthonormal projection (19). Bannadabhavi et al. developed an architecture that employs a local transformer encoder to learn community-specific node embeddings and a global transformer encoder to extract inter-community dependencies (20).

Another branch of methods for graph analysis that can be applied to connectomes is called the persistent homology (21). The idea of this approach is to analyze the evolution of topological features of a weighted network with changes of scale determined by edge weights. Topological features are global. It distinguishes them from most standard graph features that are more or less local in nature. Rathor et al. proposed a deep learning architecture that exploits the topological features of a functional connectome computed with persistent homology (22). However, the authors of this work used persistent simplicial homology. It cannot be applied to directed connectomes, which our study focuses on. There is a generalization of the persistent simplicial homology to directed graphs called the persistent homology of directed flag complex (23). Caputi et al. applied it for diagnosing schizophrenia and epilepsy (24). The disadvantage of the persistent homology of directed flag complex is that it loses some information about edge direction. Some information about edge direction in brain networks, which is lost when using this type of featurization, may negatively affect the quality of classification. Blurred magnitude homology (25), another representative of the persistent homology group of methods, is a possible option to increase the proportion of information about edge directions when constructing the feature description of an oriented network.

This paper discusses a method based on the blurred magnitude homology of directed functional connectome to classify individuals with ASD from typically developing individuals. First, a directed brain network is constructed from an fMRI scan. Then, the blurred magnitude homology of the resulting digraph is computed, and classification is performed using the blurred magnitude Betti curves.

The proposed algorithm was evaluated on the fMRI dataset (ABIDE I). It achieved rather high classification metrics and can therefore be considered a promising option for practical application.

## 2 Materials and methods

### 2.1 Data acquisition

ABIDE I, a multi-site dataset of resting-state fMRI scans from 17 international imaging sites collected by the Autism Brain Imaging Data Exchange (ABIDE) initiative (26), was employed in this study.

A subset of 871 subjects (403 patients and 468 normal controls) was used. Their scans were selected from the entire dataset after preprocessing and visual quality assessment. The preprocessed version of the ABIDE dataset provided by the Preprocessed Connectomes Projects (PCP) (27) was used with the Configurable Pipeline for the Analysis of Connectomes (C-PAC) (28) (band-pass filtering (0.01−0.1 Hz) and global signal regression were applied). The scans and their class labels (ASD/typically developing) were accessed via the function datasets.fetch_abide_pcp from the Python library Nilearn (29). To get the subset of the dataset that passed quality assessment, the argument quality_checked with the value True was used in this function.

Time series data from each subject were extracted using the Automated Anatomical Labeling (AAL; Tzourio-Mazoyer et al. (30)) probabilistic atlas. The result of this stage is *N* time series, where *N* = 116 is the number of regions of interest in the atlas.

### 2.2 Methods

The idea behind our method is based on the observation that the autism spectrum disorder (ASD) results in abnormal functional connectivity in the brain, so a possible way to diagnose it is to analyze the functional connectome. The suggested algorithm is a binary classifier: it takes as input a set of time series representing the average levels of BOLD (blood-oxygen-level-dependent) signals in brain regions and outputs a class label of the subject with two possible values: ASD or typically developing.

Our approach consists of constructing a directed functional connectome based on a set of time series, computing the topological characteristics of the resulting graph, and classifying it based on these characteristics. We chose blurred magnitude homology for featurization because it has already been studied as a digraph invariant in general (31), but there is still no research on how well it performs for connectome classification.

**Figure 1:**
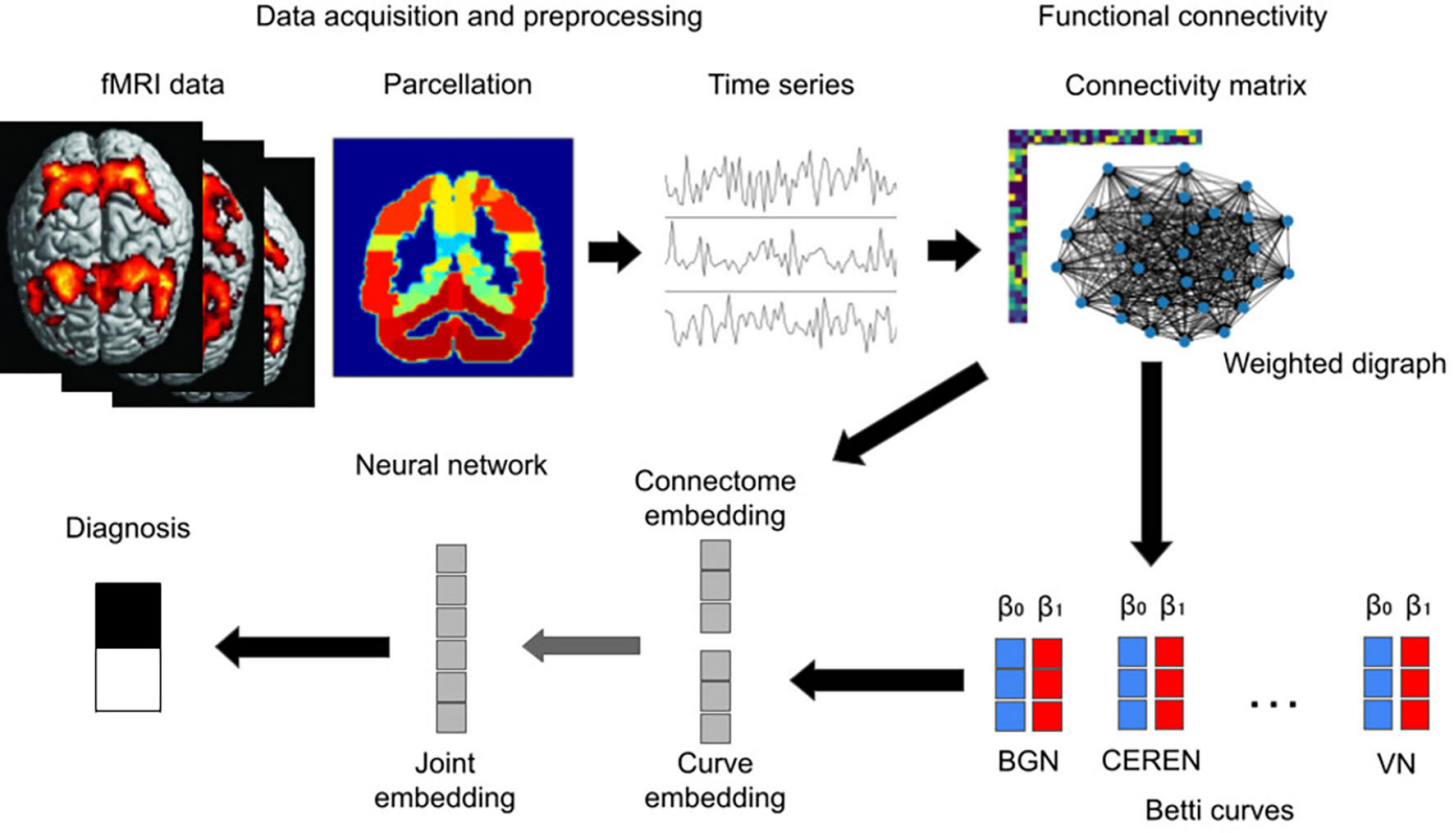
Outline of our method. Voxelwise fMRI data are parcellated to obtain multivariate time series. A directed connectivity matrix, equivalent to a directed weighted graph, is obtained using the lagged PCC of the ROI time series. Betti curves of blurred magnitude homology are computed for the directed weighted graph and passed to a neural network.

A graphical overview of the proposed method is shown in **Figure 1**.

#### 2.2.1 Method stages

The method can be split into three stages, namely:

1. Connectome construction: converting a 4D fMRI scan into a weighted directed graph.
2. Featurization: constructing a set of vectors representing topological characteristics of the graph.
3. Classification: predicting class probabilities from the computed features.

##### 2.2.1.1 Connectome construction stage

This stage consists of two steps.

First, an fMRI scan, which is a sequence of 3D voxel images, is segmented into regions of interest (ROIs) using a probabilistic atlas, and the voxel values within each ROI are averaged. The atlas used in this study is AAL (30). It consists of 116 regions.

Then, a directed fully connected connectome is constructed. Its nodes correspond to ROIs, and the weights of its edges are defined by the lagged Pearson correlation coefficient (lagged PCC). Lagged PCC measures the similarity between time-shifted versions of brain region activation time series. For two time series *x_u_* and *x_v_* of length *T*, it is defined as

(1) $$\rho_{u\to v}=\frac{1}{T-\tau -1}\sum_{t=1}^{T-\tau }\frac{\left(x_{u,t}-\mu_{u,1:T-\tau })\cdot (x_{v,t+\tau }-\mu_{v,\tau +1:T}\right)}{\sigma_{u,1:T-\tau }\cdot \sigma_{v,\tau +1:T}},$$

where $\mu_{u,1:T-\tau }$ and $\sigma_{u,1:T-\tau }$ ( $\mu_{v,\tau +1:T}$ and $\sigma_{v,\tau +1:T}$) are the sample mean and sample standard deviation of *x_u_* for time steps from 1 to *T* − *τ* (*x_v_* for time steps from *τ* + 1 to *T*, correspondingly).

In our experiments, we chose the size of the time shift *τ* = 1. It is the shortest possible delay, so the lagged PCC computed using it quantifies direct interactions between brain regions rather than indirect ones.

##### 2.2.1.2 Featurization stage

At this stage, we convert a weighted adjacency matrix of the connectome into a set of features. We suggest using an algebro-topological tool called blurred magnitude homology (BMH) (25) to numerically characterize the connectome. The characteristics themselves are called Betti curves. They are defined below.

Blurred magnitude homology is a development of the idea of magnitude homology which is more suitable for data analysis. Therefore, we start the introduction of blurred magnitude homology with magnitude homology.

###### 2.2.1.2.1 Magnitude homology

The notion of magnitude homology suitable for weighted digraphs is the magnitude homology of a quasimetric space that was originally presented in Leinster and Shulman (32). To use this homology theory for a weighted digraph, its weights must form a quasimetric. That means that if we denote by *d_u,v_* the edge weight of an edge (*u,v*), than for all nodes *v*_0_, *v*_1_, *v*_2_ the following must be true:

- Non-negativity: $d_{v_{0},v_{1}}$≥ 0.
- Identity of indiscernibles: $d_{v_{0},v_{1}}$= 0, if and only if *v*_0_ = *v*_1_.
- Triangle inequality: $d_{v_{0},v_{2}}$≤ $d_{v_{0},v_{1}}$+ $d_{v_{0},v_{2}}$.

If the edge weights of a digraph represent a measure of node similarity, they can be transformed into a quasimetric. One possible approach is discussed in Section 2.2.1.2.3.

The basic blocks of magnitude homology are tuples (finite ordered sequences) of nodes $\left(v_{0},\ldots ,v_{k}\right)$, where k≥0, such that $v_{0}\neq v_{1}\neq \ldots \neq v_{k}$. For a directed graph this notion coincides with the notion of a *k-path*, which means a path consisting of *k* edges, hence we will use the term *k*−path throughout the paper. The key numerical characteristic of a path $\left(v_{0},\ldots ,v_{k}\right)$ used in the definition of magnitude homology is the *length* $l(v_{0},\ldots ,v_{k})$ of this path. It is defined as the sum $d(v_{0},v_{1})+d(v_{1},v_{2})+\ldots +d(v_{k-1},v_{k})$.

Magnitude homology encodes the geometric properties of a quasimetric space into a family of algebraic structures *MH_k,ℓ_* indexed with a non-negative integer *k* and a non-negative real number *ℓ*. They can be groups, modules or linear spaces. For our feature extraction approach and to keep the exposition simple, we work with the case of linear spaces. These spaces are constructed from a *magnitude chain complex* that consists of:

- A family of linear spaces (*MC_k,ℓ_*)*_k_*_≥0_*_,ℓ_*_≥0_ called *spaces of magnitude chains*. The basis of *MC_k,ℓ_* is the set of all *k*-paths whose length equals *ℓ*.
- A family of linear operators (*δ_k,ℓ_*)*_k_*_≥0_*_,ℓ_*_≥0_ called *magnitude boundary operators*. *δ_k,ℓ_* is defined for an arbitrary *k*-path (*v*_0_*,…,v_k_*) of length *ℓ* by the formula

$\delta_{k,\ell }(v_{0},\ldots ,v_{k})=\sum_{i=0}^{k}{\left(-1\right)}^{i}\delta_{k,\ell }^{i}(v_{0},\ldots ,v_{k}),$

where $\delta_{k,\ell }^{i}$ transforms $\left(v_{0},\ldots ,v_{k}\right)$ into the (k−1)-path $\left(v_{0},\ldots ,{\hat{v}}_{i},\ldots ,v_{k}\right)$ obtained by dropping the node $v_{i}$ if the length of $\left(v_{0},\ldots ,{\hat{v}}_{i},\ldots ,v_{k}\right)$ equals ℓ, and to zero otherwise. From this definition we see that $\delta_{k,\ell }^{0}$ and $\delta_{k,\ell }^{k}$ always give zero, and $\delta_{k,\ell }^{i}$ for i∈{1,…,k−1} yields the non-zero tuple $\left(v_{0},\ldots ,{\hat{v}}_{i},\ldots ,v_{k}\right)$ only when $d_{v_{i-1},v_{i+1}}=d_{v_{i-1},v_{i}}+d_{v_{i},v_{i+1}}$. This situation corresponds geometrically to the case when $v_{i-1}$, $v_{i}$, and $v_{i+1}$ lie on a one line.

The subspace of a chain space mapped to zero with a boundary operator is called a *space of cycles*, and the subspace obtained as a result of applying a boundary operator is a *space of boundaries*. For magnitude homology we refer to the analogous subspaces as *space of magnitude cycles* and *space of magnitude boundaries*. A classical result in homology theory states that, for any fixed chain space, its subspace of boundaries is contained in its subspace of cycles. *MH_k,ℓ_* is defined as the quotient of the space of magnitude cycles by the space of magnitude boundaries. Consequently, *MH_k,ℓ_* is formed by non-intersecting classes of equivalent magnitude cycles, and two cycles are considered equivalent if their difference is a magnitude boundary.

To provide a clearer understanding of this rather abstract algebraic construction, we consider the most geometrically interpretable cases, *k* = 0 and *k* = 1:

- *k* = 0: *MC*_0,0_ is generated by all 1-tuples (single nodes) of the digraph, and *MC*_0_*_,ℓ_* are trivial for positive values of *ℓ*. *δ*_0,0_ is a zero operator. Therefore, the subspace of magnitude cycles of *MC*_0,0_ is *MC*_0,0_ itself, and *MH*_0,0_ coincides with *MC*_0,0_.
- *k* = 1: *MC*_1_*_,ℓ_* is generated by all directed pairs of nodes whose length equals *ℓ*. *δ*_1_*_,ℓ_* is a zero operator, so the subspace of (1*, ℓ*)-cycles is *MC*_1_*_,ℓ_* itself. *δ*_2_*_,ℓ_* maps a tuple (*v*_0_*, v*_1_*, v*_2_) of length *ℓ* to −(*v*_0_*, v*_2_) if $d_{v_{0},v_{2}}$= *ℓ*, and to zero otherwise. Consequently, the space of (1*, ℓ*)-boundaries is generated by all pairs (*u, v*) of length *ℓ* for which there exists a third node *w* not equal to *u* and *v* and satisfying the conditions *d_u,w_* = *ℓ* and *d_w,v_* = *ℓ*. Thus, *MH*_1_*_,ℓ_* corresponds to the space of pairs (*u, v*) of length *ℓ* such that every directed triangle (*u, w, v*) satisfies the strict triangle inequality *d_u, v_ < d_u,w_* + *d_w,v_*.

###### 2.2.1.2.2 Blurred magnitude homology

Blurred magnitude homology relaxes the requirement on length of paths in chain spaces by replacing the equality with non-strict inequality: blurred magnitude chain space *BMC_k,ℓ_* is generated by all the *k*-paths whose length is less or equal than *ℓ* (rather than exactly *ℓ*, as in magnitude homology).

This difference in definitions of magnitude homology and its blurred variant gives rise to significantly different homology spaces:

- *k* = 0: in blurred magnitude homology the operator *δ*_1_*_,ℓ_* is not a zero map: for a pair (*v*_0_*, v*_1_) we have *δ*_1_*_,ℓ_*(*v*_0_*, v*_1_) = (*v*_1_) − (*v*_0_). It follows that *BMH*_0_*_,ℓ_* is generated by the weakly connected components of the subgraph of the original graph that contains all nodes and all edges whose length is less or equal than *ℓ*.
- *k* = 1: the basis of subspace of cycles of *BMH*_1_*_,ℓ_* consists of 1-paths (that means directed edges). Their linear combinations form undirected cycles in the usual graph-theoretic sense. In blurred magnitude homology the boundary operator acts as *δ*_2_*_,ℓ_*(*v*_0_*, v*_1_*, v*_2_) = (*v*_1_*, v*_2_) − (*v*_0_*, v*_2_) + (*v*_0_*, v*_1_). Therefore, the elements of *BMH*_1_*_,ℓ_* correspond to classes of undirected cycles (in the graph-theoretic sense) constructed from edges whose length is less or equal than *ℓ*, and two cycles are equivalent when one can be obtained from the other by repeatedly replacing edges with a 2-path whose length is less or equal than *ℓ*.

###### 2.2.1.2.3 Edge weight transformation

As it was mentioned in the subparagraph 2.2.1.2.1, the notion of magnitude homology suitable for a weighted digraph requires its weights to form a quasimetric. The same is true for blurred magnitude homology. Lagged PCC does not satisfy any of the properties of quasimetric. It is also a measure of similarity, while a quasimetric quantifies dissimilarity. But we can turn weights into a quasimetric using the following algorithm:

1. Convert lagged PCC $\rho_{u->v}$ into a dissimilarity ${\tilde{d}}_{u,v}$ (it is not a quasimetric yet):

(2) $${\tilde{d}}_{u,v}=\sqrt{\frac{1-\rho_{u,v}}{2}}.$$

Division by 2 and taking the square root are optional but beneficial operations. The range of possible values of an expression 1 − *ρ_u,v_* is [0;2]. Division by 2 transforms it to [0;1] making this range more interpretable: the value of 1 corresponds to the case of perfect negative correlation, 0 — perfect positive correlation. Also, for an ordinary (symmetric) PCC the term $\frac{1-\rho_{u,v}}{2}$ behaves like a squared distance derived from the geometric interpretation of correlation as cosine similarity. Taking the square root transforms this into a more natural metric-like distance that aligns better with Euclidean-like distances among standardized time series. The same intuition can be extended to the case of lagged PCC.

2. Convert a dissimilarity ${\tilde{d}}_{u,v}$ into a quasimetric *d_u,v_* that is defined as the shortest weighted path distance between nodes *u* and *v* with respect to $\tilde{d}$.

For an adjacency matrix with the defined weights *d*, we can already compute our features.

###### 2.2.1.2.4 Betti curves: general case

The common way to construct a persistent homology-based feature representation of a weighted graph (denoted by *G*) is to use a set of Betti curves. This set is indexed by non-negative integers. We denote the Betti curve corresponding to index *n* by *β_n_*. Formally, a set of Betti curves is infinite. But the zeroth *β*_0_ and first *β*_1_ Betti curves are usually enough for analysis. In practice, we do need the whole curves, but only their values (called Betti numbers) at some specified points. Therefore, we can consider them vectors of Betti numbers.

The idea behind Betti curves is that we compute some topological characteristics of a weighted graph for different scales. Edge weights are assumed to represent some kind of dissimilarity between nodes — a distance in the simplest case and a quasimetric for blurred magnitude homology, for example. Technically, a view of a graph *G* at some scale *d* is a subgraph *G*_≤_*_d_* constructed on the whole set of nodes and the subset of edges with weight less or equal to *d*. A Betti number *β_n,d_* is the *n*-th topological characteristic (assuming the characteristics are indexed) of the subgraph *G*_≤_*_d_*. *A* Betti curve *β_n_* represents a set of Betti numbers *β_n,d_* for all possible weight thresholds *d*.

The idea of computing a Betti curve *β_n_* for some chosen *n*, presented above, can be formally described by the following algorithm:

1. Fix some range of weight values [*d*_min_*,d*_max_].
2. Choose some set of points *d*_min_ = *d*_0_*< d*_1_*<… < d_T_*_−__1_*< d_T_* = *d*_max_ within this range.
3. For all *w* in (*d*_0_*, d*_1_*,…,d_T_*), compute the Betti number *β_n,d_* of the subgraph *G*≤*_d_*.

As a final feature representation of the weighted graph *G*, we have a vector of numbers ( $\beta_{n,d_{0}},\beta_{n,d_{1}},\ldots ,\beta_{n,d_{T}}$).

###### 2.2.1.2.5 Betti curves: blurred magnitude homology

For blurred magnitude homology, *β*_0_*_,ℓ_* is the number of weakly connected components of a subgraph of the connectome that includes all nodes and only those edges with weight less or equal to *ℓ*. The first Betti number *β*_1_*_,ℓ_* counts the number of non-intersecting classes of undirected cycles composed of edges with length at most *ℓ*. Two cycles belong to the same class if one of them can be obtained from the other by applying a sequence of specific deformations. Here a deformation is understood as replacing a directed edge (*v*_0_*, v*_2_), which belongs to the considered cycle and also to the directed triangle (*v*_0_*, v*_1_*, v*_2_) such that $d_{v_{0},v_{1}}$+ $d_{v_{1},v_{2}}$≤ *ℓ*, with a two-path (*v*_0_, *v*_1_, *v*_2_). Two cycles representing different classes are called independent.

For our case, we choose the range [*d*_min_, *d*_max_] to be equal [0,1] for the zeros Betti curve *β*_0_, and [0,2] — for the first Betti curve *β*_1_. The choice of the range for *β*_0_ is motivated by the fact that this range coincides with the range of values of the proposed distance function *d*. For *β*_1_, the range corresponds to values obtained as sums of two distances associated with directed triangles from the given definition. In practice, a uniform grid over the chosen range is fixed.

**Figure 2:**
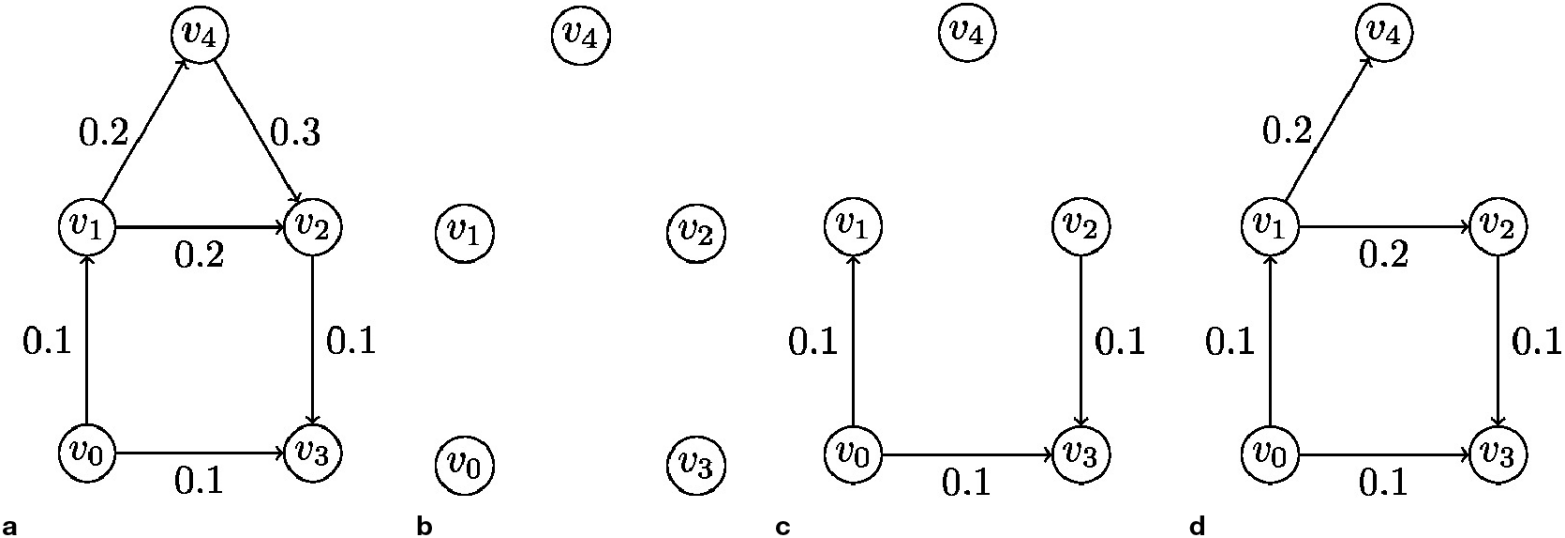
Example of a 5-node weighted digraph and its subgraphs obtained by adding edges in ascending order of weights. **(a)** The original graph G. **(b)** The subgraph *G*_≤0_. **(c)** The subgraph *G*_≤0.1_. **(d)** The subgraph *G*_≤0.2_.

To provide a better understanding of the proposed feature representation, we present here an example of its computation for a small digraph:

Example 1 (an example of blurred magnitude Betti curves): We compute blurred magnitude Betti curves for a quasimetric space defined by a five-node digraph illustrated in **Figure 2a**. Actually, a quasimetric space corresponds to a complete digraph. However, the process of constructing Betti curves is easier to understand using an example of a non-complete digraph, as its visualization looks simpler. Therefore, we can formally assign an infinite weight to all directed edges that are not present in our digraph.

Now we apply the steps of the algorithm for computing Betti numbers:

1. Choice of a weight range: we choose the range [0, 0.5] of weights for computing Betti curves since below 0 and above 0.5 they will not change.
2. Choice of a grid on the weight range: we find the grid (0, 0.1, 0.2, 0.3, 0.4, 0.5) convenient for this example.
3. Computing the 0-th and the 1-st Betti numbers for a set of subgraphs:

- *G*_≤0_ is a graph with five nodes and no edges (see **Figure 2b**). It contains five weakly connected components, each corresponding to a single node, so *β*_0,0_ = 5. It contains no cycles, so *β*_1,0_ = 0.
- *G*_≤0.1_ is a graph with the set of edges {(*v*_0_*, v*_1_),(*v*_0_*, v*_3_),(*v*_2_*, v*_3_)} (see **Figure 2c**). It contains two weakly connected components: {*v*_0_*,v*_1_*,v*_2_*,v*_3_} and {*v*_4_}; so, *β*_0,0.1_ = 2. It contains no cycles, so *β*_1,0.1_ = 0.
- *G*_≤0.2_ is a graph with the set of edges {(*v*_0_*,v*_1_),(*v*_1_*,v*_2_),(*v*_2_*,v*_3_),(*v*_0_*,v*_3_),(*v*_1_*,v*_4_)} (see **Figure 2d**). It contains one weakly connected component that coincides with the entire set of nodes, so *β*_0,0.2_ = 1. It contains one undirected cycle (*v*_0_*,v*_1_*,v*_2_*,v*_3_*,v*_0_) and no ordered triples of length less than or equal to 0.2, so *β*_1,0.2_ = 1.
-
$G_{\leq 0.3}$ corresponds to the original graph. It contains one weakly connected component, that coincides with the entire set of nodes, so $\beta_{1,0.3}$= 1. It contains two independent (with respect to deformation via ordered triples) undirected cycles: for example, (*v*_0_*,v*_1_*,v*_2_*,v*_3_*,v*_0_) and (*v*_1_*,v*_2_*,v*_4_*,v*_1_). There are no ordered triples of length less than or equal to 0.3, so *β*_1,0.3_ = 2.
-
$G_{\leq 0.4}$corresponds to the original graph. The situation is the same as for $G_{\leq 0.3}$, so *β*_0,0.4_ = 1 and *β*_1,0.4_ = 2.
-
$G_{\leq 0.5}$ corresponds to the original graph. It contains one weakly connected component, that coincides with the entire set of nodes, so *β*_0,0.5_ = 1. It contains two independent (with respect to deformation via ordered triples) undirected cycles. Here, it is better to consider cycles (*v*_0_*,v*_1_*,v*_2_*,v*_3_*,v*_0_) and (*v*_0_*,v*_1_*,v*_4_*,v*_2_*,v*_3_*,v*_0_), as there is 1 ordered triple (*v*_1_*,v*_2_*,v*_4_) of length less than or equal to 0.5. With such a choice of independent cycles, it is clear that we can obtain the longer cycle (*v*_0_*,v*_1_*,v*_4_*,v*_2_*,v*_3_*,v*_0_) by replacing the directed edge (*v*_1_*,v*_2_) with the ordered triple (*v*_1_*,v*_2_*,v*_4_), so *β*_1,0.5_ = 2 − 1 = 1.

**Figure 3:**
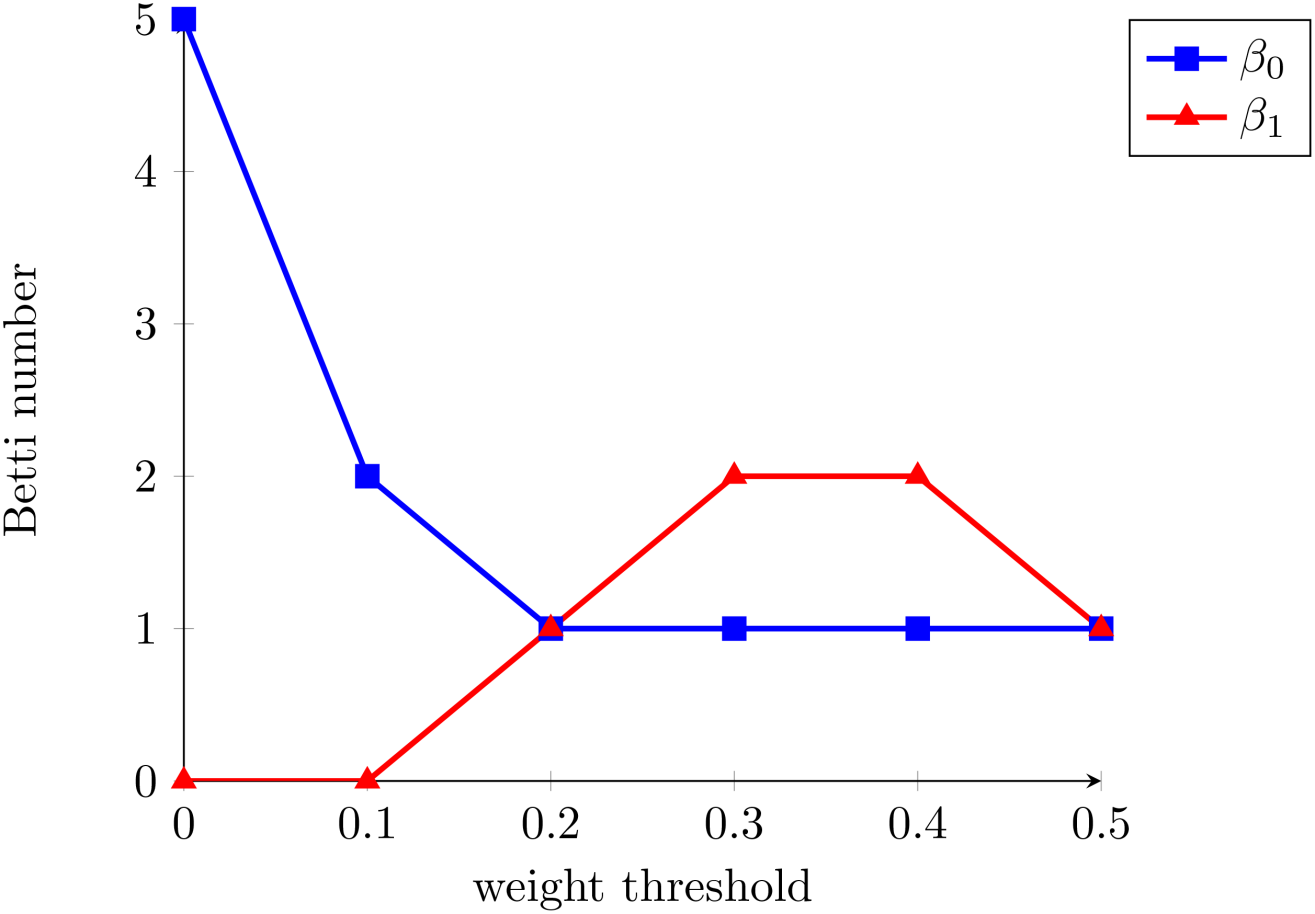
Example of blurred magnitude Betti curves.

The Betti curves for the considered example are presented in **Figure 3**. According to the featurization algorithm that we propose, the feature representation of the graph from the example consists of two vectors: *β*_0_ = (5,2,1,1,1,1) and *β*_1_ = (0,0,1,2,2,1).

Betti numbers are computed as ranks of boundary operator matrices (for a detailed explanation, please see (21)). The matrices involved are quite large, so computing Betti curves for the entire connectome can be time-consuming and even intractable in practice for large brain atlases. Therefore, we decided to split all ROIs from the atlas that we use (AAL) into nine functional subnetworks, as suggested in Hu et al. (33) and compute Betti curves for each subnetwork separately. The final feature representation of connectome consists of 18 curves: the zeroth and the first Betti curve for each of the nine subnetworks.

##### 2.2.1.3 Classification stage

We chose a neural network with two backbones as the architecture of the classifier for our method. One backbone is used to construct the embedding of the connectome, and another is used for the Betti curves. Then, the outputs of these two backbones are concatenated and passed to an MLP for classification.

More formally,

(3) $$\beta_{0}^{\text{joint}}=\text{CONCAT}(\beta_{0}^{\text{BGN}},\beta_{0}^{\text{CEREN}},\beta_{0}^{\text{DAN}},\beta_{0}^{\text{DMN}},\beta_{0}^{\text{FPN}},\beta_{0}^{\text{LN}},\beta_{0}^{\text{SMN}},\beta_{0}^{\text{VAN}},\beta_{0}^{\text{VN}}),$$

(4) $$\beta_{1}^{\text{joint}}=\text{CONCAT}(\beta_{1}^{\text{BGN}},\beta_{1}^{\text{CEREN}},\beta_{1}^{\text{DAN}},\beta_{1}^{\text{DMN}},\beta_{1}^{\text{FPN}},\beta_{1}^{\text{LN}},\beta_{1}^{\text{SMN}},\beta_{1}^{\text{VAN}},\beta_{1}^{\text{VN}}),$$

(5) $$x_{\text{Betti}}=\text{CONCAT}(\beta_{0}^{\text{joint}},\beta_{1}^{\text{joint}}),$$

(6) $$e_{\text{connectome}}={\text{EMB}}_{\text{connectome}}(x_{\text{connectome}}),$$

(7) $$e_{\text{Betti}}={\text{EMB}}_{\text{Betti}}(x_{\text{Betti}}),$$

(8) $$e_{\text{joint}}=\text{CONCAT}(e_{\text{connectome}},e_{\text{Betti}}),$$

(9) $${\text{cls}}_{\text{pred}}=\text{CLF}(e_{\text{joint}}),$$

where $x_{\text{connectome}}$—input features for a connectome, $x_{\text{Betti}}—$input features for Betti curves, $e_{\text{connectome}}—$an embedding for a connectome, $e_{\text{Betti}}—$an embedding for Betti curves,

CONCAT—an operation of concatenation of vectors,
EMB_connectome_ and EMB_Betti_—backbones with an architecture of an MLP for constructing an embedding of connectome and Betti curves, correspondingly,
CLF—a classifier with an architecture of an MLP.

The abbreviations for functional subnetworks are as follows: BGN (basal ganglia network), CEREN (cerebellar network), DAN (dorsal attention network), DMN (default mode network), FPN (frontoparietal network), LN (limbic network), SMN (sensorimotor network), VAN (ventral attention network), and VN (visual network).

#### 2.2.2 Implementation

fMRI BOLD signals, preprocessed with the C-PAC pipeline, were obtained using the Nilearn library (29). Directed connectomes based on lagged PCC and blurred magnitude Betti curves were both computed with the NumPy library (34). The implementation of the Dijkstra algorithm from the NetworkX library (35) was used to compute shortest path lengths in a functional connectome.

The classification algorithms were implemented using the Scikit-Learn (36) and PyTorch (37) libraries.

## 3 Results

### 3.1 Experiments

To assess the quality of the proposed method, experiments were carried out on digraphs constructed from fMRI scans. The argmaximum of F-score on the validation sample was chosen as the threshold for binarization of the classifier outputs.

#### 3.1.1 Model comparison

For comparison of our approach with other methods, we chose the following ones in the role of baselines:

- Support vector machine with radial basis function kernel (RBF-SVM) trained on ROI BOLD signals averaged over time steps, that means vectors of length equal to the number of ROIs.
- Random forest (RF) trained on ROI BOLD signals averaged over time steps.
- MLP trained on ROI BOLD signals averaged over time steps.
- RBF-SVM trained on graph features.
- RF trained on graph features.
- MLP trained on graph features.
- MLP trained on a vectorized weighted adjacency matrix of a lagged PCC-based functional connectome.

According to the literature review, we selected the following graph features: in-degree (116 features), out-degree (116 features), betweenness centrality (116 features), pagerank centrality (116 features), global characteristic path length (one feature), global clustering coefficient (that means averaged local clustering coefficients; one feature), local efficiency (116 features), global efficiency (one feature), flow coefficient (116 features), assortativity (one feature) and transitivity (one feature), totaling 701 features. Graph features were computed for a binarized adjacency matrix of the connectome based on lagged PCC. Binarization was performed by selecting a certain proportion of edges showing the strongest connectivity.

Among the more advanced methods used for comparison, we considered ASD-DiagNet (16), an autoencoder with classifier; CNNG (7), a combination of a convolutional neural network and a gated recurrent unit; and NF-GAT (17), and a graph neural network with a multi-head attention mechanism. Moreover, we compared the performance of the method exploiting Betti curves of the persistent homology of directed flag complex instead of blurred magnitude homology and an MLP trained exclusively on Betti curves of blurred magnitude homology (BMH-MLP) and Betti curves of persistent homology of directed flag complex (DPH-MLP) without incorporating the connectivity matrix. ASD-DiagNet and NF-GAT were trained on connectomes computed using the Pearson correlation coefficient (PCC) and CNNG—on BOLD signals averaged over ROIs.

The AdamW optimizer was used to train all neural networks. Cross-entropy loss was employed in the role of a loss function.

The hyperparameters of the algorithms were chosen using grid search with fivefold cross-validation and five different random seeds. For MLP trained using Betti curves of blurred magnitude homology, the best hyperparameters are one hidden layer of size 128 with ReLU activation function, a dropout of 0.5, a learning rate of 1e-5, a weight decay of 1e-6, and 100 training epochs. For MLP trained using Betti curves of persistent homology of directed flag complex, the best hyperparameters are one hidden layer of size 128 with ReLU activation function, a dropout of 0.5, a learning rate of 3e-6, a weight decay of 1e-6, and 100 training epochs. For the network using a connectivity matrix and Betti curves (of both blurred magnitude homology and persistent homology of directed flag complex), the best hyperparameters are one fully connected layer of size 128 with ReLU activation function for both backbones, a one-layer fully connected classification head, a dropout of 0.5 for the classification head, a learning rate of 1e-5, a weight decay of 1e-5, and 50 training epochs. For RBF-SVM trained on an averaged BOLD signal, the best hyperparameter is a regularization parameter of 1. For RF trained on an averaged BOLD signal, the best hyperparameter is 100 estimators. For MLP trained on an averaged BOLD signal, the best hyperparameters are one hidden layer of size 128 with ReLU activation function, a dropout of 0.5, a learning rate of 1e-5, a weight decay of 1e-6, and 200 training epochs. For RBF-SVM trained on graph features, the best hyperparameters are a regularization parameter of 1 and a binarization threshold for the adjacency matrix of 0.7. For RF trained on graph features, the best hyperparameters are 300 estimators and a binarization threshold for the adjacency matrix of 0.6. For MLP trained on graph features, the best hyperparameters are one hidden layer of size 128 with ReLU activation function, a dropout of 0.5, a learning rate of 1e-5, a weight decay of 1e-6, 100 training epochs, and a binarization threshold for the adjacency matrix of 0.8. For MLP trained on a connectivity matrix, the best hyperparameters are one hidden layer of size 128 with ReLU activation function, a dropout of 0.5, a learning rate of 1e-5, a weight decay of 1e-6, and 50 training epochs. For ASD-DiagNet, the best hyperparameters are an autoencoder hidden dimension of 200, a dropout for the autoencoder of 0.3, a dropout for the classification head of 0.5, a learning rate of 1e-4, a weight decay of 1e-6, and 100 training epochs. For NF-GAT, the best hyperparameters are 32 hidden channels, 3 attention heads, a learning rate of 1e-5, a weight decay of 1e-6, and 50 training epochs.

In our experiments, we used a grid of size 256 for all Betti curves of the functional subnetworks. The grid size was optimized as a hyperparameter. For further details, please refer to Subsubsection 3.1.4.

F-score, accuracy, precision and sensitivity calculated from the model outputs binarized using a threshold that gives the maximum of the former metric was chosen to evaluate the classifiers. Comparison of the tested algorithms by these metrics is given in the **Table 1**. We denoted our method as BMH-Net and the method based on the persistent homology of directed flag complex as DPH-Net. The best metric value is highlighted in bold.

**Table 1:** Metric values in the experiments; the threshold for binarization of outputs for every algorithm is selected in the maximum of the corresponding F-score.

Algorithm	F-score	Accuracy	Precision	Sensitivity
BOLD RBF-SVM	0.634±0.003	0.465±0.007	0.464±0.004	0.999±0.007
BOLD MLP	0.634±0.003	0.472±0.017	0.467±0.008	0.990±0.024
BOLD RF	0.636±0.006	0.476±0.021	0.469±0.010	0.991±0.017
Graph features RBF-SVM	0.643±0.012	0.509±0.048	0.487±0.028	0.953±0.060
Graph features RF	0.643±0.010	0.511±0.041	0.487±0.022	0.951±0.052
Graph features MLP	0.646±0.010	0.516±0.040	0.490±0.023	0.951±0.054
CNNG	0.633±0.003	0.465±0.005	0.464±0.003	$\underset{0.003}{0.999\pm }$
NF-GAT	0.633±0.003	0.463±0.003	0.463±0.003	1.0±0.000
DPH-MLP	0.635±0.004	0.476±0.032	0.470±0.020	0.984±0.048
BMH-MLP	0.643±0.009	0.513±0.048	0.491±0.030	0.947±0.073
DPH-Net	0.661±0.018	0.578±0.060	0.534±0.049	0.882±0.075
ASD-DiagNet	0.665±0.031	$\underset{0.053}{0.621\pm }$	$\underset{0.048}{0.567\pm }$	0.811±0.051
Connectome MLP	$\underset{0.021}{0.671\pm }$	0.615±0.062	0.566±0.058	0.845±0.089
BMH-Net (ours)	0.674±0.022	0.622±0.065	0.573±0.061	0.840±0.085

The best metric value is highlighted in bold.

We conducted statistical testing using the paired Wilcoxon signed-rank test to assess whether differences in performance metrics between BMH-Net (our method) and other algorithms are statistically significant. The p-values, adjusted using the Holm correction for multiple comparisons, are presented in **Table 2**. Using a significance level of 0.05, our method demonstrates statistically significant superiority in F-score over all compared models. For accuracy and precision, the differences with ASD-DiagNet are not statistically significant. However, the observed differences in these metrics, while not reaching statistical significance at the chosen threshold, suggest a consistent trend in favor of our method. Additionally, we performed a separate comparison between BMH-MLP and DPH-MLP. The p-values for this comparison are as follows: for F-score — ≪ 1*e* − 3, for accuracy — 0.002, for precision — 0.001, and for recall — 0.008. Based on these results and using a significance level of 0.05, we conclude that Betti curves of blurred magnitude homology better distinguish connectomes of different classes in terms of F-score, accuracy, and precision, but perform worse in terms of recall.

**Table 2:** *P*-values with Holm correction for the null hypothesis that the median metric for BMH-Net and the paired algorithm are the same.

Algorithm	F-score	Accuracy	Precision	Recall
BOLD RBF-SVM	≪1e−3	≪1e−3	≪1e−3	≪1e−3
BOLD MLP	≪1e−3	≪1e−3	≪1e−3	≪1e−3
BOLD RF	≪1e−3	≪1e−3	≪1e−3	≪1e−3
Graph features RBF-SVM	≪1e−3	≪1e−3	≪1e−3	0.001
Graph features RF	≪1e−3	≪1e−3	≪1e−3	0.001
Graph features MLP	≪1e−3	≪1e−3	≪1e−3	0.001
CNNG	≪1e−3	≪1e−3	≪1e−3	≪1e−3
NG-GAT	≪1e−3	≪1e−3	≪1e−3	≪1e−3
DPH-MLP	≪1e−3	≪1e−3	≪1e−3	≪1e−3
BMH-MLP	≪1e−3	≪1e−3	≪1e−3	0.006
DPH-Net	≪1e−3	0.004	0.012	0.070
ASD-DiagNet	0.042	0.761	0.751	0.122
Connectome MLP	0.004	0.003	0.004	0.010

#### 3.1.2 Model analysis

**Figure 4:**
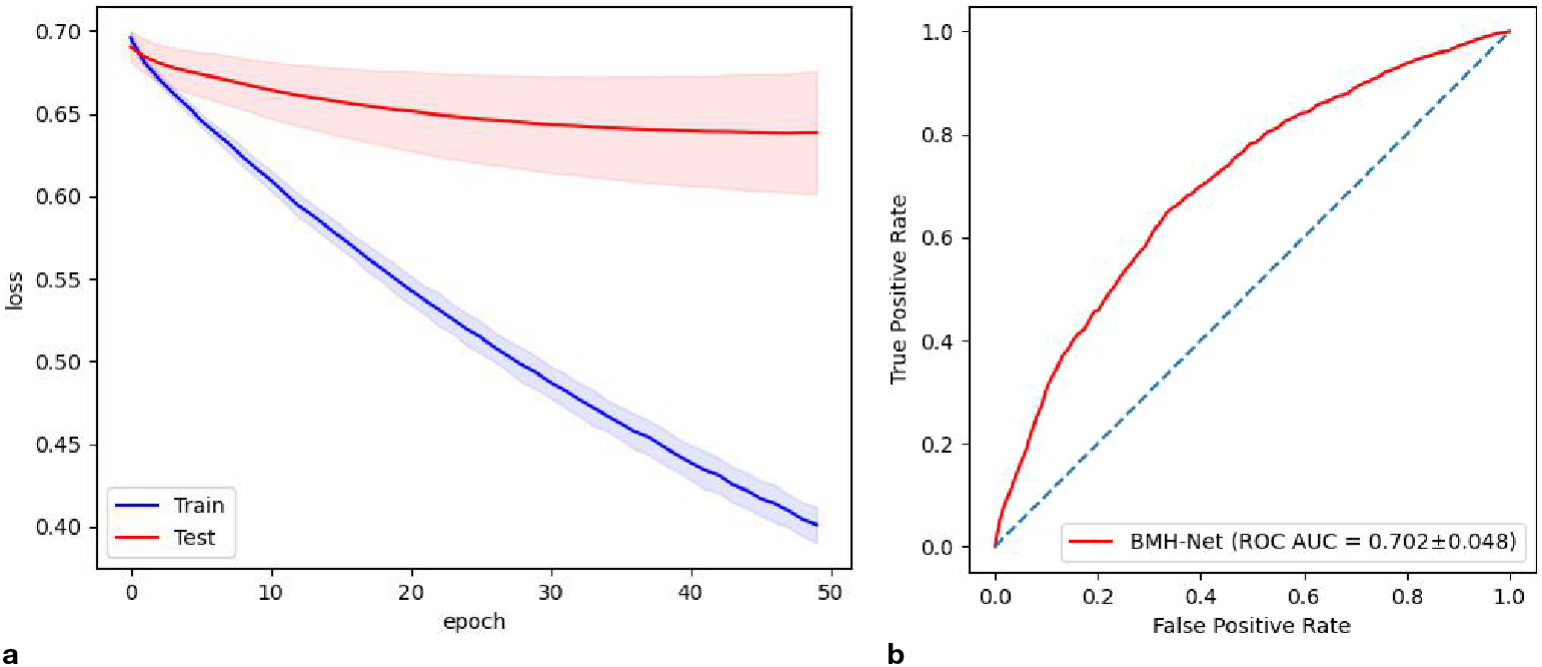
**(a)** The plot of loss function for BMH-Net. **(b)** The ROC curve for BMH-Net.

The plot of the loss function for BMH-Net is presented in **Figure 4a**, and the ROC curve is in **Figure 4b**.

#### 3.1.3 Subnetwork feature importance

We evaluated the level of importance of the Betti curves of each functional subnetwork on making a prediction by our model. The algorithm is as follows:

1. For each pair (random seed, cross-validation split), compute the gradient of the model output with respect to Betti curves for all objects of the validation subset.
2. For each validation subset, estimate the importance of each individual value of each Betti curve as the average of absolute values of the gradients over all objects of this subset.
3. Estimate the importance of Betti curves for the whole functional subnetwork as the sum of the importances of the values of these Betti curves.

**Figure 5:**
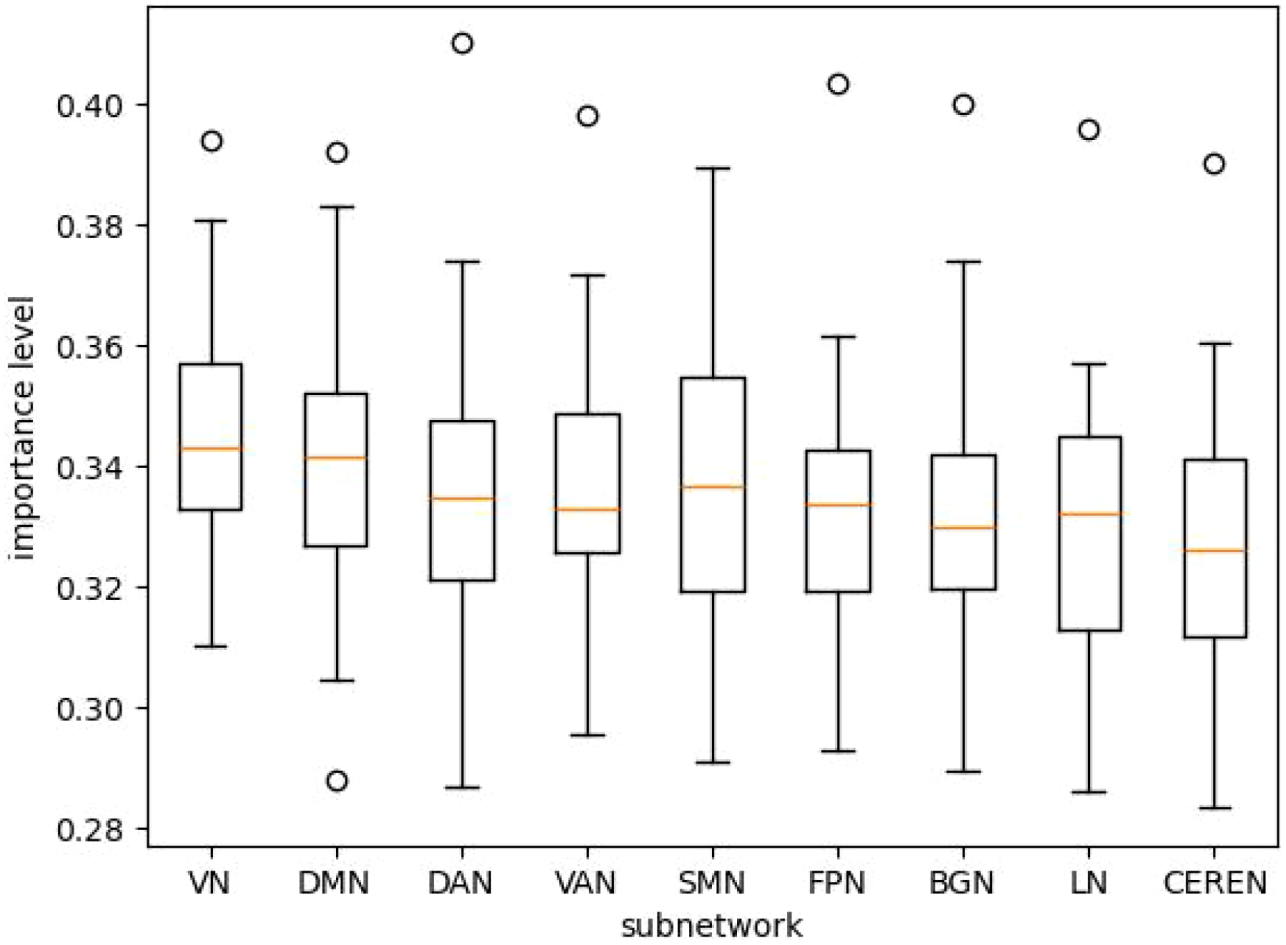
Subnetwork importance boxplots for BMH-Net.

The computed importances aggregated over all pairs (random seed, cross-validation split) are presented in **Table 3**, and the box plots are in **Figure 5**.

**Table 3:** Subnetwork feature importances for BMH-Net.

Subnetwork	Importance
VN	0.346 ± 0.020
DMN	0.340 ± 0.023
DAN	0.337 ± 0.025
VAN	0.337 ± 0.022
SMN	0.336 ± 0.023
FPN	0.334 ± 0.022
BGN	0.333 ± 0.023
LN	0.331 ± 0.023
CEREN	0.328 ± 0.023

#### 3.1.4 Effect of Betti curves grid size on classification performance

To select an appropriate grid size for blurred magnitude Betti curves, we evaluated three options: 128, 256, and 512. The classification performance metrics for our method BMH-Net, corresponding to the grid sizes for Betti curves set to these values, are presented in **Table 4**. We observed that the F-score monotonically decreases as the grid size increases, while sensitivity is the highest at the largest grid size tested (512). Accuracy and precision both peak at the grid size of 256. Considering these results, we chose 256 as a balanced compromise grid size that optimizes accuracy and precision while accepting trade-offs in F-score and sensitivity.

**Table 4:** Metric values of BMH-Net with different grid sizes for Betti curves; the threshold for binarization of outputs is selected in the maximum of the corresponding F-score.

Grid size	F-score	Accuracy	Precision	Sensitivity
128	**0**.**676** ± **0**.**022**	0.618 ± 0.067	0.569 ± 0.061	0.856 ± 0.095
256	0.674 ± 0.022	**0**.**622** ± **0**.**065**	**0**.**573** ± **0**.**061**	0.840 ± 0.085
512	0.670 ± 0.018	0.599 ± 0.058	0.550 ± 0.051	**0**.**875** ± **0**.**082**

The best metric value is highlighted in bold.

#### 3.1.5 Computation time of blurred magnitude Betti curves

The computation time of blurred magnitude Betti curves was measured on 10 randomly selected connectomes from the ABIDE I dataset. The average runtimes (mean ± SD) were 31.664 ± 5.396 s for a grid size of 128, 62.245 ± 9.668 s for 256, and 126.165 ± 18.358 s for 512. All calculations were performed on a computer equipped with an AMD Ryzen 7 5700U processor and 16 GB of RAM.

#### 3.1.6 Relationship between functional subnetwork alterations in ASD and blurred magnitude Betti curves

**Figure 6:**
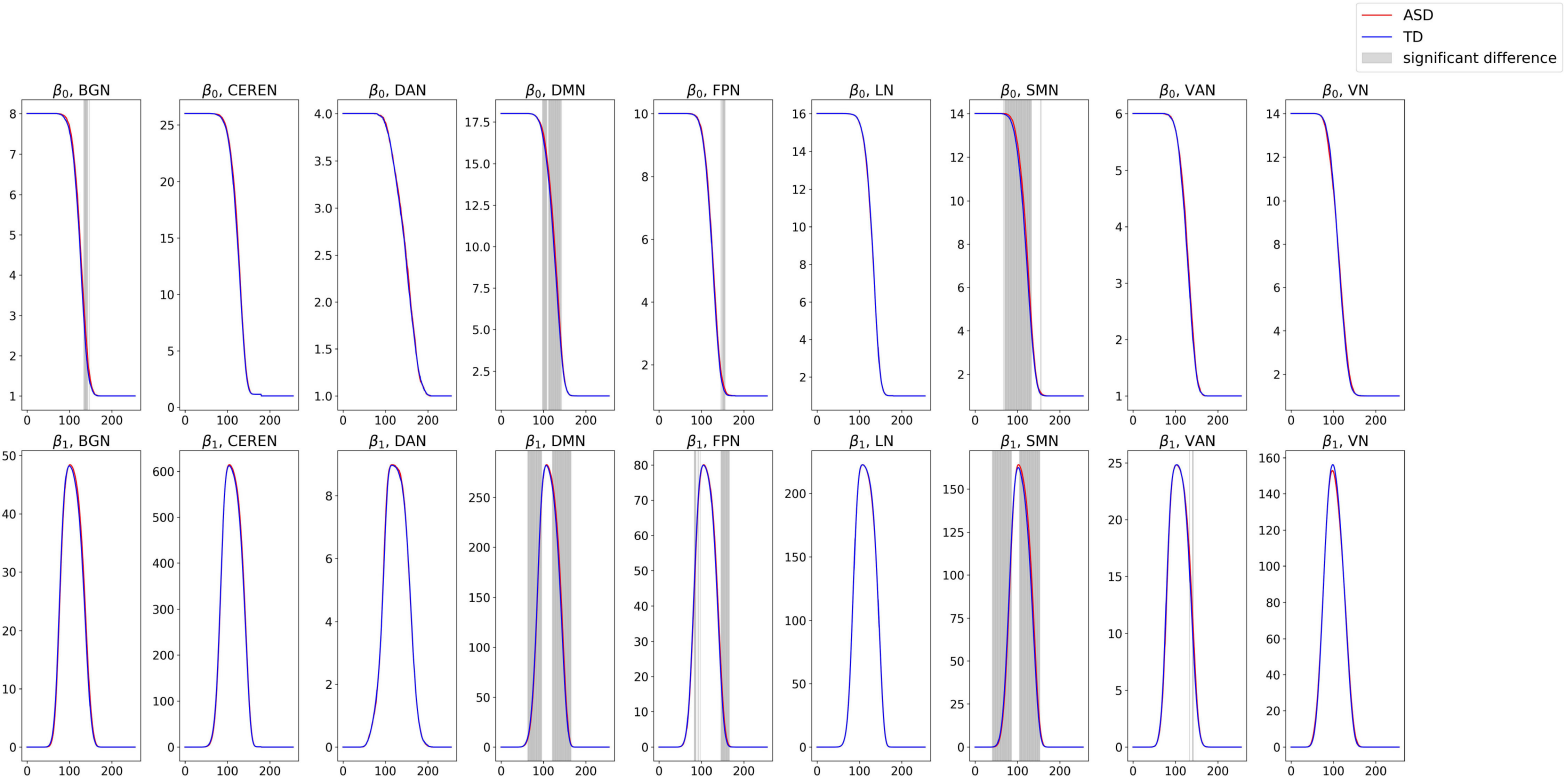
Blurred-magnitude Betti curves for functional subnetworks averaged over ASD and TD groups for the ABIDE dataset.

To explore the relationship between blurred magnitude Betti curve values of functional subnetworks and functional connectivity alterations in ASD reported in prior neuroscientific studies, we conducted independent-samples *t*-tests for each edge weight threshold value across all 18 Betti curves computed for the ABIDE dataset. The statistical significance was first tested with an uncorrected *p*-value of 0.05, and then Holm correction was applied to adjust for multiple comparisons. This analysis tested whether the average Betti number differs significantly between ASD patients and typically developing individuals across all edge weight thresholds within each subnetwork. The average Betti curves for the ASD and TD groups, with significant threshold values marked by gray vertical lines, are shown in **Figure 6**. Statistically significant differences emerged in the basal ganglia, default mode network, frontoparietal network, sensorimotor network, and ventral attention network.

These findings agree with existing studies that have reported changes in functional connectivity within these functional subnetworks in ASD—for example, abnormal basal ganglia connectivity has been linked to motor and cognitive symptoms in ASD, as shown by (38). The default mode network (DMN) shows altered connectivity related to social difficulties in ASD, as reported by Padmanabhan et al. (39). Changes in the frontoparietal network (FPN) and ventral attention network (VAN), which are important for executive function and attention, have been likewise found in ASD (e.g., (40)). The sensorimotor network (SMN) also shows connectivity differences related to sensorimotor problems in ASD, as documented by (41).

These observations suggest that the blurred magnitude Betti curves may capture meaningful functional and clinical changes in brain network topology in ASD. While previous studies have reported abnormalities in certain functional subnetworks, our approach helps to bring together these separate findings across multiple subnetworks, providing a more comprehensive view of functional connectome alterations in ASD.

## 4 Discussion

The paper presents a new type of topological features of a directed functional connectome, namely, Betti curves of blurred magnitude homology, and proposes a way in which they can be used to diagnose ASD using machine learning models. The effectiveness of the approach was evaluated on the task of classifying real fMRI images of ASD and typically developing individuals.

The classification metrics calculated in the experiments reveal that the classifier using the suggested feature description outperforms the existing approaches on some performance metrics (F-score, accuracy, and precision) but underperforms some of the existing methods in sensitivity. The higher values of the metrics for the same classifier trained on the Betti curves of blurred magnitude homology compared to the ones of the persistent homology of directed flag complex—the most straightforward way to generalize classical persistent homology to the case of a directed network—show that blurred magnitude homology distinguishes functional connectomes of ASD and typically developing individuals better than the persistent homology of directed flag complex. The fact that BMH-Net achieved higher metrics than the MLP working with vectorized connectome proves that blurred magnitude Betti curves carry additional information about a functional connectome that the classifier used cannot extract from the adjacency matrix itself.

It is also worth noting that the proposed approach is applicable to the diagnosis of any neurological disorder that alters a functional connectivity of brain regions. In particular, our method could be extended in future work to analyze blurred magnitude homology-based features of brain networks in other neuropsychiatric disorders, such as attention deficit hyperactivity disorder. Such extensions represent promising directions for further exploring the applicability of the developed technique to a broader range of conditions.

Since ASD in adulthood is one of the possible causes of early brain aging, this work also contributes to methods for postponing the onset and progression of cognitive decline.

The limitation of our approach is that the algorithm for computing blurred magnitude Betti curves does not scale well to large brain networks because of its rather high time complexity. Developing an efficient algorithm similar to the one used for the persistent simplicial homology is one of the directions of future work. For BMH it will require a data structure that enables efficient storage and manipulation of simplicial sets, which appear directly in its definition, and an algorithm for computing persistent homology that is specifically optimized for chain complexes constructed from those simplicial sets.

## Data Availability

Publicly available datasets were analyzed in this study. This data can be found here: http://fcon_1000.projects.nitrc.org/indi/abide/abide_I.html.

## References

[1] Dickinson A Jeste S Milne E . Electrophysiological signatures of brain aging in autism spectrum disorder. Cortex. (2022) 148:139–51. doi: 10.1016/j.cortex.2021.09.022, PMID: 35176551 PMC11813168

[2] Sigar P Kathrein N Gragas E Kupis L Uddin LQ Nomi JS . Age-related changes in brain signal variability in autism spectrum disorder. Mol Autism. (2025) 16:8. doi: 10.1186/s13229-024-00631-3, PMID: 39923093 PMC11806755

[3] Rane P Cochran D Hodge SM Haselgrove C Kennedy DN Frazier JA . Connectivity in autism: a review of mri connectivity studies. Harvard Rev Psychiatry. (2015) 23:223–44. doi: 10.1097/HRP.0000000000000072, PMID: 26146755 PMC5083037

[4] Hull JV Dokovna LB Jacokes ZJ Torgerson CM Irimia A Van Horn JD . Resting-state functional connectivity in autism spectrum disorders: a review. Front Psychiatry. (2017) 7:205. doi: 10.3389/fpsyt.2016.00205, PMID: 28101064 PMC5209637

[5] Mohammad-Rezazadeh I Frohlich J Loo SK Jeste SS . Brain connectivity in autism spectrum disorder. Curr Opin Neurol. (2016) 29:137–47. doi: 10.1097/WCO.0000000000000301, PMID: 26910484 PMC4843767

[6] Ahammed MS Niu S Ahmed MR Dong J Gao X Chen Y . Darkasdnet: classification of asd on functional mri using deep neural network. Front Neuroinformatics. (2021) 15:635657. doi: 10.3389/fninf.2021.635657, PMID: 34248531 PMC8265393

[7] Jiang W Liu S Zhang H Sun X Wang S-H Zhao J . Cnng: A convolutional neural networks with gated recurrent units for autism spectrum disorder classification. Front Aging Neurosci. (2022) 14:948704. doi: 10.3389/fnagi.2022.948704, PMID: 35865746 PMC9294312

[8] Ke H Wang F Bi H Ma H Wang G Yin B . Unsupervised deep frequency-channel attention factorization to non-linear feature extraction: A case study of identification and functional connectivity interpretation of parkinson’s disease. Expert Syst Appl. (2024) 243:122853. doi: 10.1016/j.eswa.2023.122853

[9] Wang F Ke H Cai C . Deep wavelet self-attention non-negative tensor factorization for non-linear analysis and classification of fmri data. Appl Soft Computing. (2025) 182:113522. doi: 10.1016/j.asoc.2025.113522

[10] Wang F Ke H Ma H Tang Y . Deep wavelet temporal-frequency attention for nonlinear fmri factorization in asd. Pattern Recognition. (2025) 165:111543. doi: 10.1016/j.patcog.2025.111543

[11] Wang F Ke H Tang Y . Fusion of generative adversarial networks and non-negative tensor decomposition for depression fmri data analysis. Inf Process Manage. (2025) 62:103961. doi: 10.1016/j.ipm.2024.103961

[12] Chaitra N Vijaya PA Deshpande G . Diagnostic prediction of autism spectrum disorder using complex network measures in a machine learning framework. Biomed Signal Process Control. (2020) 62. doi: 10.1016/j.bspc.2020.102099

[13] Kazeminejad A Sotero RC . Topological properties of resting-state fmri functional networks improve machine learning-based autism classification. Front Neurosci. (2019) 12:1018. doi: 10.3389/fnins.2018.01018, PMID: 30686984 PMC6335365

[14] Mijalkov M Volpe G Pereira JB . Directed brain connectivity identifies widespread functional network abnormalities in parkinson’s disease. Cereb Cortex. (2022) 32:593–607. doi: 10.1093/cercor/bhab237, PMID: 34331060 PMC8805861

[15] Khazaee A Ebrahimzadeh A Babajani-Feremi A . Classification of patients with mci and ad from healthy controls using directed graph measures of resting-state fmri. Behav Brain Res. (2017) 322:339–50. doi: 10.1016/j.bbr.2016.06.043, PMID: 27345822

[16] Eslami T Mirjalili V Fong A Laird AR Saeed F . Asd-diagnet: a hybrid learning approach for detection of autism spectrum disorder using fmri data. Front Neuroinformatics. (2019) 13:70. doi: 10.3389/fninf.2019.00070, PMID: 31827430 PMC6890833

[17] Liu S Liang B Wang S Li B Pan L Wang S-H . Nf-gat: A node feature-based graph attention network for asd classification. IEEE Open J Eng Med Biol. (2023) 5:428–33. doi: 10.1109/OJEMB.2023.3267612, PMID: 38899023 PMC11186657

[18] Li X Zhou Y Dvornek N Zhang M Gao S Zhuang J . Braingnn: Interpreta ble brain graph neural network for fmri analysis. Med Image Anal. (2021) 74. doi: 10.1016/j.media.2021.102233, PMID: 34655865 PMC9916535

[19] Kan X Dai W Cui H Zhang Z Guo Y Yang C . Brain network transformer. In: Koyejo S Mohamed S Agarwal A Belgrave D Cho K Oh A , editors. Advances in neural information processing systems. Red Hook, NY, USA: Curran Associates, Inc (2022). p. 25586–99.

[20] Bannadabhavi A Lee S Deng W Ying R Li X . (2023). Community-aware transformer for autism prediction in fmri connectome, in: International conference on medical image computing and computer-assisted intervention, Medical Image Computing and Computer Assisted Intervention -- MICCAI 2023, pp. 287–97. Cham, Switzerland: Springer Nature Switzerland AG.

[21] Edelsbrunner H Harer J . Computational topology: an introduction. Providence: American Mathematical Society (2010).

[22] Rathore A Palande S Anderson JS Zielinski BA Fletcher PT Wang B . (2019). Autism classification using topological features and deep learning: a cautionary tale, in: International Conference on Medical Image Computing and Computer-Assisted Intervention, Medical Image Computing and Computer Assisted Intervention -- MICCAI 2019, pp. 736–44. Cham, Switzerland: Springer International Publishing., PMID: 10.1007/978-3-030-32248-9_82 PMC7390646 32728675

[23] Lütgehetmann D Govc D Smith J Levi R . Computing persistent homology of directed flag complexes. Algorithms. (2020) 13:19. doi: 10.3390/a13010019

[24] Caputi L Pidnebesna A Hlinka J . Promises and pitfalls of topological data analysis for brain connectivity analysis. NeuroImage. (2021) 238. doi: 10.1016/j.neuroimage.2021.118245, PMID: 34111515

[25] Otter N . Magnitude meets persistence. homology theories for filtered simplicial sets. arXiv preprint arXiv. (2018) 1807:01540. doi: 10.48550/arXiv.1807.01540

[26] Di Martino A Yan C-G Li Q Denio E Castellanos F Alaerts K . The autism brain imaging data exchange: Towards large-scale evaluation of the intrinsic brain architecture in autism. Mol Psychiatry. (2013) 19:659–67. doi: 10.1038/mp.2013.78, PMID: 23774715 PMC4162310

[27] Craddock C Benhajali Y Carlton C Francois C Evans A Jakab A . The neuro bureau preprocessing initiative: open sharing of preprocessed neuroimaging data and derivatives. Front Neuroinformatics. (2013) 7:2013.09.00041. doi: 10.3389/conf.fninf.2013.09.00041

[28] Craddock C Sikka S Cheung B Khanuja R Ghosh SS Yan C . Towards automated analysis of connectomes: The configurable pipeline for the analysis of connectomes (c-pac). Front Neuroinformatics. (2013) 42:2013.09.00042. doi: 10.3389/conf.fninf.2013.09.00042

[29] Abraham A Pedregosa F Eickenberg M Gervais P Mueller A Kossaifi J . Machine learning for neuroimaging with scikit-learn. Front Neuroinformatics. (2014) 8:14. doi: 10.3389/fninf.2014.00014, PMID: 24600388 PMC3930868

[30] Tzourio-Mazoyer N Landeau B Papathanassiou D Crivello F Etard O Delcroix N . Automated anatomical labeling of activations in spm using a macroscopic anatomical parcellation of the mni mri single-subject brain. NeuroImage. (2002) 15:273–89. doi: 10.1006/nimg.2001.0978, PMID: 11771995

[31] Ivanov S . Nested homotopy models of finite metric spaces and their spectral homology. arXiv preprint arXiv. (2023) 2312:11878. doi: 10.48550/arXiv.2312.11878

[32] Leinster T Shulman M . Magnitude homology of enriched categories and metric spaces. Algebraic Geometric Topology. (2021) 21:2175–221. doi: 10.2140/agt.2021.21.2175

[33] Hu B Guan X Zhai H Han X Hu C Gong J . Cognitive and cortical network alterations in pediatric temporal lobe space-occupying lesions: an fmri study. Front Hum Neurosci. (2024) 18:1509899. doi: 10.3389/fnhum.2024.1509899, PMID: 39717148 PMC11663916

[34] Harris CR Millman KJ van der Walt SJ Gommers R Virtanen P Cournapeau D . Array programming with numpy. Nature. (2020) 585:357–62. doi: 10.1038/s41586-020-2649-2, PMID: 32939066 PMC7759461

[35] Hagberg AA Schult DA Swart PJ . Exploring network structure, dynamics, and function using networkx. In: Varoquaux G Vaught T Millman J , editors. Proceedings of the 7th python in science conference (SciPy2008) Austin Texas: SciPy (2008). p. 11–5.

[36] Pedregosa F Varoquaux G Gramfort A Michel V Thirion B Grisel O . Scikit-learn: Machine learning in python. J Mach Learn Res. (2011) 12:2825–30. doi: 10.1145/2786984.2786995

[37] Paszke A Gross S Massa F Lerer A Bradbury J Chanan G . Pytorch: An imperative style, high-performance deep learning library. In: Wallach H Larochelle H Beygelzimer A d’Alché-Buc F Fox E Garnett R , editors. Advances in neural information processing systems, vol. 32 . Red Hook, NY, USA: Curran Associates, Inc (2019). p. 8024–35.

[38] Shang J Shen E Yu Y Jin A Wang X Xiang D . Relationship between abnormal intrinsic functional connectivity of subcortices and autism symptoms in high-functioning adults with autism spectrum disorder. Psychiatry Research: Neuroimaging. (2024) 337:111762. doi: 10.1016/j.pscychresns.2023.111762, PMID: 38043369

[39] Padmanabhan A Lynch CJ Schaer M Menon V . The default mode network in autism. Biol Psychiatry: Cogn Neurosci Neuroimaging. (2017) 2:476–86. doi: 10.1016/j.bpsc.2017.04.004, PMID: 29034353 PMC5635856

[40] Yerys BE Tunc¸ B Satterthwaite TD Antezana L Mosner MG Bertollo JR . Functional connectivity of frontoparietal and salience/ventral attention networks have independent associations with co-occurring attention-deficit/hyperactivity disorder symptoms in children with autism. Biol Psychiatry: Cogn Neurosci Neuroimaging. (2019) 4:343–51. doi: 10.1016/j.bpsc.2018.12.012, PMID: 30777604 PMC6456394

[41] Anderson JS Druzgal TJ Froehlich A DuBray MB Lange N Alexander AL . Decreased interhemispheric functional connectivity in autism. Cereb Cortex. (2011) 21:1134–46. doi: 10.1093/cercor/bhq190, PMID: 20943668 PMC3077433

